# Association between optical coherence tomography based retinal microvasculature characteristics and myocardial infarction in young men

**DOI:** 10.1038/s41598-018-24083-x

**Published:** 2018-04-04

**Authors:** Robert Kromer, Eike Tigges, Nargis Rashed, Inga Pein, Maren Klemm, Stefan Blankenberg

**Affiliations:** 1 Department of Ophthalmology, University Medical Center Hamburg-Eppendorf, Hamburg, Germany; 2 Department of General and Interventional Cardiology, University Heart Center, University Medical Center Hamburg-Eppendorf, Hamburg, Germany

## Abstract

Incident myocardial infarction (MI) is a leading cause of adult mortality in the United States. However, because MI has a relatively low incidence in the young population, little information exists on the disease in younger adults. Because the retina has the unique quality that its vasculature is readily and noninvasively visible, the retina is frequently studied to evaluate correlations between vessels and cardiovascular diseases. In the current study, we evaluated the retinal microvasculature of patients who had experienced an MI before 50 years of age (n = 53 subjects) and age- and sex-matched patients who had not experienced an MI (n = 53 patients). We used circular optical coherence tomography (OCT) scans to image peripapillary venules and arterioles. The diameter of each vessel was measured and the respective arterial-venous ratio (AVR) was calculated. We did not detect any significant differences between MI and control subjects in retinal vessel calibre or AVR.

## Introduction

Mortality from coronary heart disease (CHD) has significantly declined in the developed world over the past several decades. However, it remains the leading cause of adult mortality in the United States^1^. The CHD classification describes disease from subclinical atherosclerosis to acute coronary events, including myocardial infarction (MI). Even though CHD and its subsequent events primarily occur in patients over the age of 45 years, an age threshold has not been defined^2^. In fact, sudden cardiac death (predominantly due to CHD) is the most common cause of sudden death in adults under 40 years of age^3^. Therefore, CHD and its sequelae constitute an important health issue. This is especially true in younger patients who have a 15-year mortality rate of 30% (for patients < 40 years of age)^4^ and whose active lifestyles are at risk. Unfortunately, the mortality rate significantly increased to 45% for subjects who had experienced a previous MI^4^. These findings highlight the need to develop a risk stratification, which could lead to earlier diagnoses in high risk patients. However, CHD in the younger adult population is less common than CHD in older populations. As a result, comparatively little information exists on the condition in younger populations.

The retina has the unique characteristic that its vasculature can be directly visualized and its microcirculation can be noninvasively imaged in a matter of seconds. Retinal microvasculature analyses can quantify parameters related to vessel structure and function^5^. Additionally, blood vessels in the eye and heart are exposed to similar intrinsic- and extrinsic-influencing factors^6^. Moreover, the central retinal artery and its direct branches, generally evaluated with ophthalmoscopy, are true arteries because they have three vessel wall layers (intima, media, and adventitia). They also closely resemble small arteries in other organs, including the heart and brain^7^. Thus, changes in retinal vasculature characteristics may be early warning signs of ocular and/or systemic disease. In support of this theory, atherosclerotic changes in retinal arteries, characterized by thickening of the vessel wall, lipid deposition in the intima, and calcification of larger arteries, is strongly associated with atherosclerotic changes in the coronary arteries^8,9^. This is particularly true in young patients, in whom arteriolar narrowing and arteriovenous nicking correlate with cardiovascular risk factors (e.g., increased blood pressure)^10^ and inflammation markers^11^. Moreover, morphological features of retinal vessels (e.g., length, width, tortuosity, and branching pattern), have been used for screening and evaluation of various cardiovascular diseases associated with cardiovascular risk factors^12,13^.

Spectral-domain optical coherence tomography (SD-OCT) is an established imaging method that noninvasively produces high-resolution, cross-sectional images of biological tissue^14^. In ophthalmology, this technology is used as a diagnostic tool because it allows the retinal layers to be visualized and the complicated retinal cytoarchitecture to be examined *in vivo*^15^. Vessel diameters can be indirectly measured using OCT images because vessels characteristically create shadows, or dark columns, beneath them (light does not penetrate the blood vessel so underlying tissues cannot be imaged). Vessels can be characterized as arteries or veins based on their appearance on simultaneously collected fundus images. The arterial-venous ratio (AVR) is an established marker of retinal vessel morphology changes and can be calculated using OCT vessel measurements.

In the current study, we investigated potential changes in the calibre of retinal vessels and the AVR in subjects who had experienced an MI before the age of 50 years. More specifically, we evaluated central retinal vessels in the peripapillary region using SD-OCT. All fundus images were obtained using confocal-scanning laser ophthalmoscopy (cSLO). Measurements were then compared to those made in a control group made up of healthy, age- and sex-matched subjects.

## Results

This study included 53 male subjects who experienced an MI before the age of 50 years and 53 age-matched healthy male subjects who had not experienced an MI. Subjects had experienced an MI at an average age of 40.7 ± 5.2 years and, at the time of enrolment, mean subject age was 57.3 ± 10.6 and 57.9 ± 10.5 years in the MI and control groups, respectively. In the MI group, the average time between MI and study enrolment was 16.6 ± 10.8 years. There was a significant correlation between subject age in the MI and control groups (r = 0.98, p < 0.0001) and no significant difference using a paired t-test (p = 0.07), indicating that the two groups had been successfully age-matched.

Intra-subject comparisons between eyes indicated no significant differences (p > 0.05). Subjects in the MI group had a central retinal arterial equivalent (CRAE-6) of 47.6 ± 2.2 and 46.9 ± 1.9 μm, a central retinal venous equivalent (CRVE-6) of 57.1 ± 2.0 and 56.6 ± 2.2 μm, and an AVR of 0.83 ± 0.04. and 0.83 ± 0.05 in the right eye and left eye, respectively. Subjects in the control group had a CRAE-6 of 47.5 ± 2.0 and 46.9 ± 1.6 μm, a CRVE-6 of 57.0 ± 1.8 and 56.8 ± 2.0 μm, and an AVR of 0.83 ± 0.04 and 0.83 ± 0.03 for the right and left eyes, respectively. There was no significant difference between eyes in any parameter examined in either the MI or control group (all p > 0.05, Table 1).

**Table 1:** Retinal microvasculature parameters in healthy control subjects and in those who experienced a myocardial infarction (MI) at a young age (<50 years). All parameters were measured in both eyes. Measured values in the right eye and left eyes were compared using two-tailed paired t-tests.

	Right eye	Left eye	p
Healthy subjects			
CRAE-6 (µm)	47.5 ± 2.0	46.9 ± 1.6	0.16
CRVE-6 (µm)	57.0 ± 1.8	56.8 ± 2.0	0.64
AVR	0.83 ± 0.04	0.83 ± 0.03	0.43
MI subjects			
CRAE-6 (µm)	47.6 ± 2.2	46.9 ± 1.9	0.09
CRVE-6 (µm)	57.1 ± 2.0	56.6 ± 2.2	0.18
AVR	0.83 ± 0.04	0.83 ± 0.05	0.82

Central retinal arterial equivalent (CRAE-6); Central retinal venous equivalent (CRVE-6); Arterial-venous ratio (AVR).

**Figure 1:**
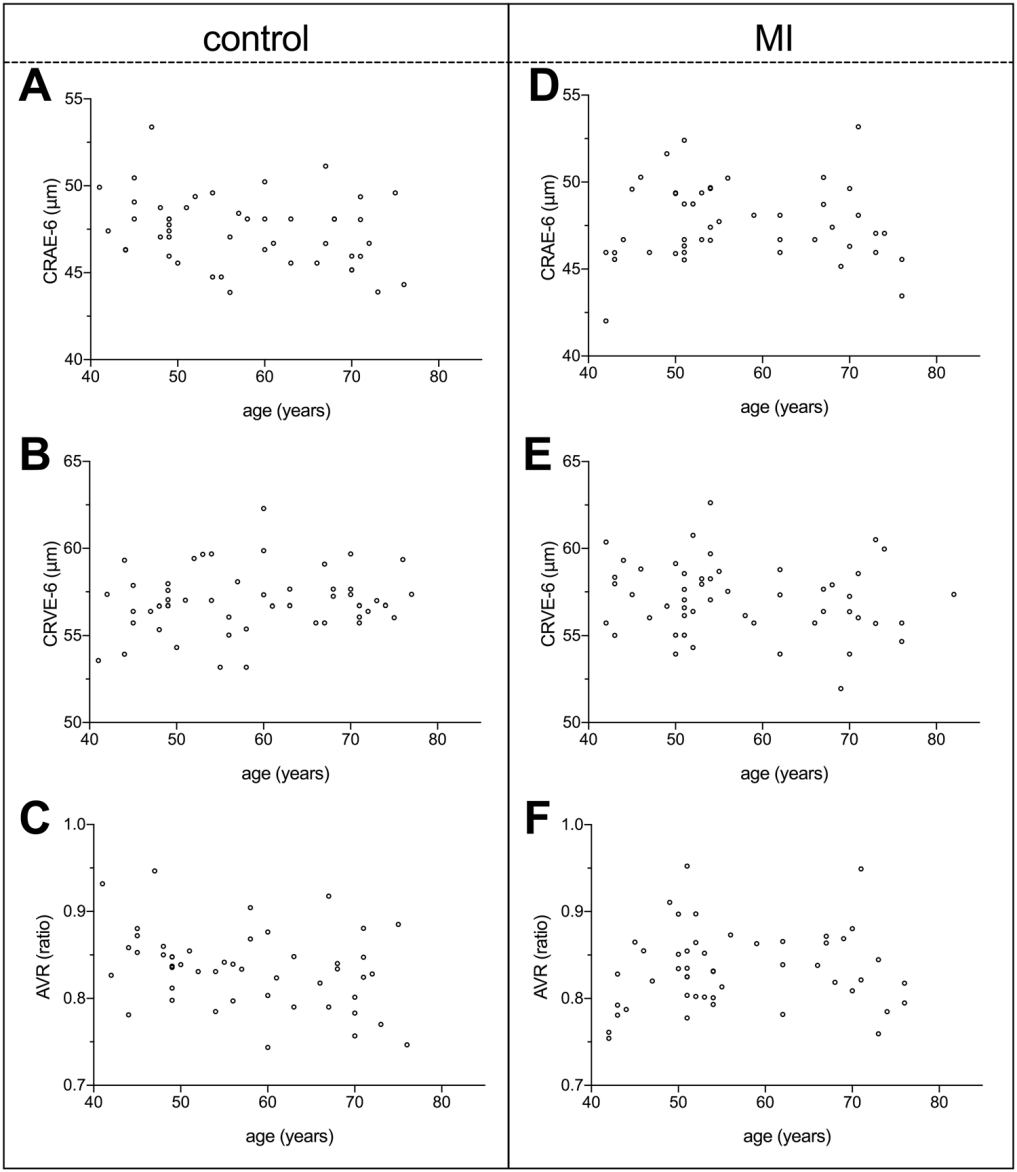
Correlation plots showing the influence of age on examined retinal microvasculature parameters in healthy control subjects (**A**–**C**) and in those who experienced a myocardial infarction (MI) at a young age (<50 years, **D**–**F**). The central retinal arterial equivalent (CRAE-6, **A** and **D**), central retinal venous equivalent (CRVE-6, B and E), and arteriovenous ratio (AVR, c and f) were examined. Age was not significantly correlated with any retinal microvasculature parameter examined (all p > 0.05). All data shown were collected from the right eye of study subjects.

**Figure 2:**
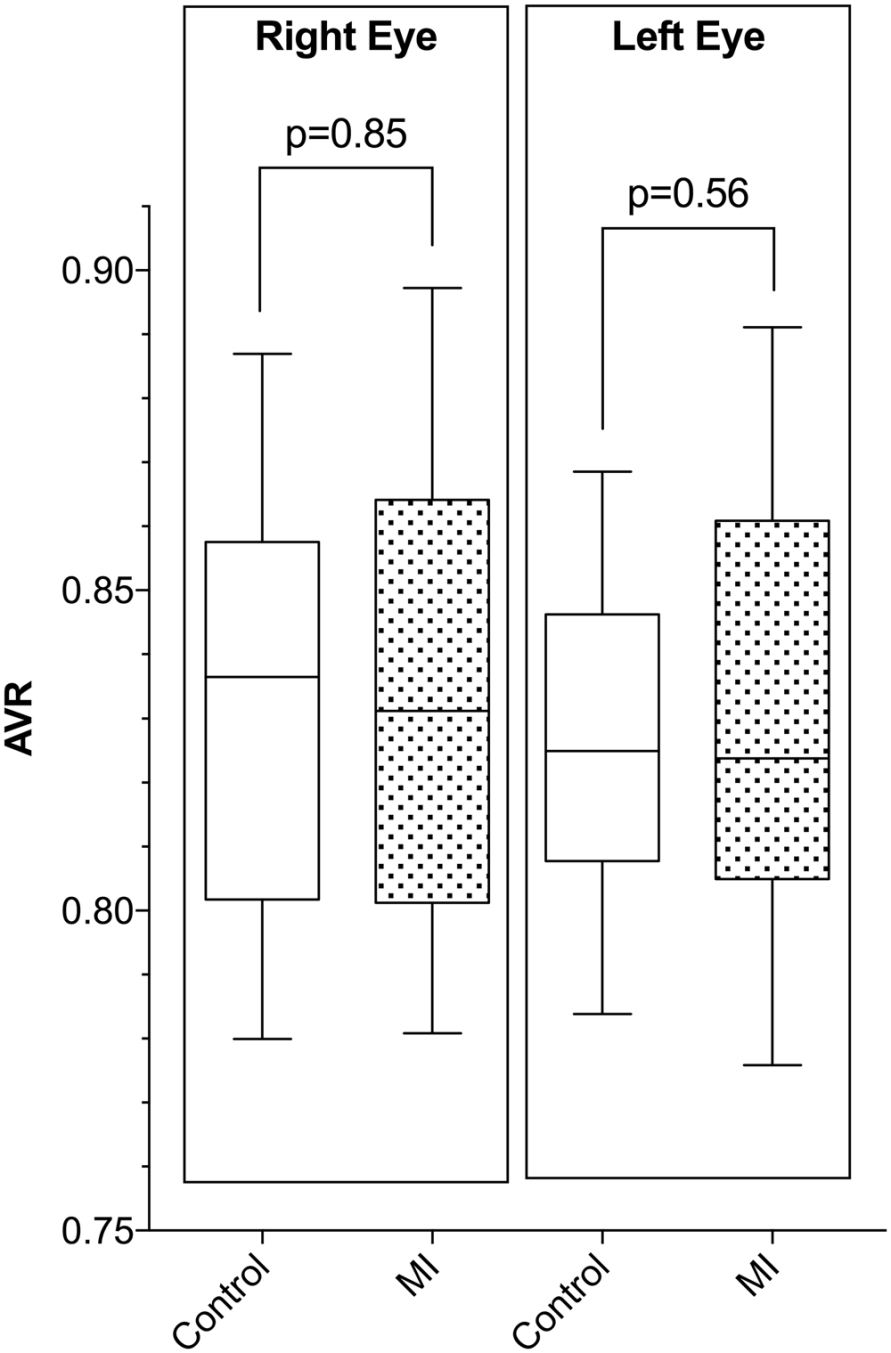
The arteriovenous ratio (AVR) in the right eye and left eye for healthy subjects and for those who experienced a myocardial infarction (MI) at a young age (<50 years). The AVR was not significantly different between groups in either the right eye (mean of differences = 0.002; p = 0.85) or the left eye (mean of differences = −0.005; p = 0.56). Statistical significance was tested using a paired t-test. Vertical columns represent 25–75% percentile with indicated mean and error bars represent 10–90% percentile.

Correlations between age and CRAE-6 (right eye: p = 0.92, left eye: p = 0.72), CRVE-6 (right eye: p = 0.29, left eye: p = 0.17), and AVR (right eye: p = 0.50, left eye: p = 0.37) were not statistically significant (Fig. 1). Furthermore, AVR was not significantly different between the MI and control groups in either the right eye (mean of differences = 0.002, p = 0.85) or the left eye (mean of differences = −0.005, p = 0.56; Fig. 2, Table 2). A post-study power analysis indicated that the study was adequately powered (99%) to detect the smallest average difference in AVR of 0.039 and 0.034 (two-tailed, α = 0.05) in the right eye and left eye, respectively.

**Table 2:** Retinal microvasculature parameters in healthy control subjects and in those who experienced a myocardial infarction (MI) at a young age (<50 years). All parameters were measured in both eyes. Measured values were compared between groups using two-tailed paired t-tests.

	Healthy subjects	MI subjects	p
Right eye			
CRAE-6 (µm)	47.5 ± 2.0	47.6 ± 2.2	0.87
CRVE-6 (µm)	57.0 ± 1.8	57.1 ± 2.0	0.82
AVR	0.83 ± 0.04	0.83 ± 0.04	0.85
Left eye			
CRAE-6 (µm)	46.9 ± 1.6	46.9 ± 1.9	0.81
CRVE-6 (µm)	56.8 ± 2.0	56.6 ± 2.2	0.58
AVR	0.83 ± 0.03	0.83 ± 0.05	0.56

Central retinal arterial equivalent (CRAE-6); Central retinal venous equivalent (CRVE-6); Arterial-venous ratio (AVR).

## Discussion

Our results indicate, with a power of 99%, that men who experienced an MI before the age of 50 years did not have changes in the retinal microvasculature, as examined with vessel calibre and AVR. However, coronary microvascular dysfunction is not likely an isolated process, but part of a systemic one^16,17^. This theory is supported by the finding that retinal arteriolar narrowing may be an early indicator of microvascular damage caused by aging, hypertension, and/or other processes and that it reflects intimal thickening and medial hyperplasia, hyalinization, and sclerosis^18^. Because similar pathological processes occur in the coronary arterioles of patients with hypertension^19,20^, changes that occur in retinal arterioles may offer insight into changes that occur in the systemic microcirculation. Additionally, AVR, an index of retinal arteriolar narrowing, has been shown to be correlated with long-term average blood pressure^21^ and to be a marker of inflammation and endothelial dysfunction^22^. Furthermore, retinal vascular architecture changes could offer insight into subclinical CHD. Previous studies have shown that a smaller retinal arteriolar caliber is associated with left ventricular concentric remodeling, identified using cardiac MRI^11,23^. This association remained significant even after adjusting for traditional risk factors.

Our study only examined male subjects and our results may have been different if women had also been included in our study cohort. The Atherosclerosis Risk in Communities Study included 9648 patients and provided evidence that a smaller AVR was linked to an increased risk of incident CHD and MI in women, but not in men^24^. Comparable gender differences in the association between AVR and CHD were also observed in the Blue Mountains Eye Study, Beaver Dam Eye Study, and Cardiovascular Health Study^25–27^. A recent meta-analysis by McGeechan *et al*.^28^ (n > 22,000 subjects) concluded that both wider venules and narrower arterioles were associated with an increased risk of CHD in women, but not in men. It is important to note that all of the above-mentioned studies only included participants who were middle-aged or older. Therefore, it is likely that microvascular dysfunction plays a more integral role in the pathogenesis of CHD in women than in men^29^. These gender differences may result from the influence of hormones or other substances that inhibit atherosclerosis development. Furthermore, these findings suggest that microvascular processes are the main contributors to CHD development^30^, as supported by the finding that revascularization procedures have better outcomes in men than in women^31^.

A prior study showed that middle-aged and older men with a history of MI do not have an altered retinal microvasculature^24^. Our study differed in that as it only included patients who experienced an MI before the age of 50. Our study adds to the literature because little is known about baseline clinical characteristics and CHD risk factors in the young MI population^32^. Prior studies that examined younger CHD or MI patients used an age cut-off of 40 or 45 years to distinguish between young and “nonyoung” patients^33–38^. These investigations found that young patients were more likely to be male, to be a smoker, and to have a family history of CHD. In agreement, young men with hypertension are significantly less likely to develop CHD than women in the same age group^32,39^. In summary, epidemiological data, clinical findings, and the current results support the hypothesis that microvascular changes play little to no role in the development of CHD and MI in young men. Klein *at al*. showed in a study involving 8772 adults that the pathogenesis of retinal arteriolar changes differs^22^. They demonstrated that retinal arteriolar changes like focal narrowing, generalized narrowing, changed AVR or arteriovenous nicking seem to be related to hypertension, inflammatory factors and endothelial dysfunction. However, clinical and subclinical atherosclerosis was seen independently to these retinal arteriolar changes. They suggested that atherosclerosis, as a disease of larger arteries, provides an independent prediction for ischemic diseases of the heart and other organs. In our study, we did not find any retinal arteriolar changes in terms of altered AVR in men who experienced an MI before the age of 50 years. Therefore, early-onset atherosclerosis might play a major role in the development of CHD and MI in this population rather than hypertension, inflammatory factors or endothelial dysfunction.

Previous research demonstrated a significant correlation between AVR and age, which was not replicated in our study^40^. This might be attributed to the low patient number compared to epidemiological studies and the young age of the included participants. The study excluded patients with both high alcohol intake and body mass index >30 kg/m^2^. These might have effects on the microvasculature and could lead to an altered AVR^41,42^.

Our study had several limitations. First, patients were not examined immediately after the MI had occurred. In some cases, subjects had experienced the MI decades earlier. This may have influenced our results because arteriolar narrowing may have been alleviated with antihypertensive therapy^43^. However, antihypertensive therapy does not affect retinal venules^43,44^. Second, additional data regarding cardiovascular risk factors were not obtained. Third, our sample size adequately powered the current study, but it was small compared to epidemiological and population studies. Our study also had several strengths. This is the first study to evaluate the retinal vasculature in subjects who had experienced an MI at a young age. Additionally, SD-OCT is an objective, accurate, and established method for evaluating the retinal vessels. Lastly, only images that met the OSCAR-IB consensus criteria for retinal OCT quality were included in data analyses.

In summary, we demonstrated that men who had experienced an MI before 50 years of age do not have associated changes in the retinal microvasculature. Further studies are needed to identify factors that can feasibly be used as screening tools to determine which patients have a high-risk for MI. This information can then be used to support clinical decision making.

## Methods

The study protocol was reviewed and approved by the Ethics Committee of the Hamburg Medical Council. All study conduct adhered to the tenets of the Declaration of Helsinki (7^th^ revision, 64^th^ Meeting, Fortaleza, Brazil) and followed Good Clinical Practices (GCPs). Written informed consent was obtained from each participant before study evaluations were performed.

### Study subjects

Subjects who were already participating in the University Heart Center “MI Young Study” at the University Medical Centre Hamburg-Eppendorf were prospectively recruited for participation in the current study. The ongoing “MI Young Study” began in 2015 and is evaluating the risk profile of young (<50 years of age) MI patients using routinely-performed cardiac diagnostics, echocardiography, and genetic profiling. Age- and sex-matched healthy control subjects were prospectively recruited from the Department of Ophthalmology at the University Medical Center Hamburg-Eppendorf. Subjects were selected based on careful review of medical histories and records, with particular attention given to retinal and cardiovascular disease.

Subjects in the MI Young Study who met all of the following ophthalmic criteria were considered for study participation: (i) best-corrected logMAR visual acuity of 0.3 or better, (ii) spherical refractive error within ±5.0 dioptres (D), and (iii) cylindrical refractive error within ±2.0 D. Subjects were excluded if any of the following were present: (i) intense alcohol abuse (more than 40 g of alcohol daily), (ii) body mass index >30 kg/m^2^, (iii) intraocular pressure ≥21 mmHg, (iv) anterior ischemic optic neuropathy, or (v) congenital optic nerve abnormality. All participants in both the MI and control groups had a physiological or medication controlled systemic blood pressure within normal limits (within the range of 100–130 millimetres mercury (mmHg) systolic and 60–85 mmHg diastolic). Healthy control subjects with cardiovascular diseases, including angina, myocardial infarction, stroke, heart failure, hypertensive heart disease, rheumatic heart disease, cardiomyopathy, heart arrhythmia, congenital heart disease, valvular heart disease, carditis, aortic aneurysms, peripheral artery disease, thromboembolic disease and venous thrombosis, were excluded from the study.

### Study evaluations

Study participants underwent a thorough ophthalmic examination, including: (i) slit lamp-assisted biomicroscopy (anterior and posterior segment thru non-dilated pupils) to identify any ocular pathology, (ii) non-contact tonometry (Nidek Tonometer NT-530) to identify undetected glaucoma, and (iii) SD-OCT imaging to collect study measurements (SPECTRALIS; Heidelberg Engineering, Heidelberg, Germany).

#### Optical coherence tomography

**Figure 3:**
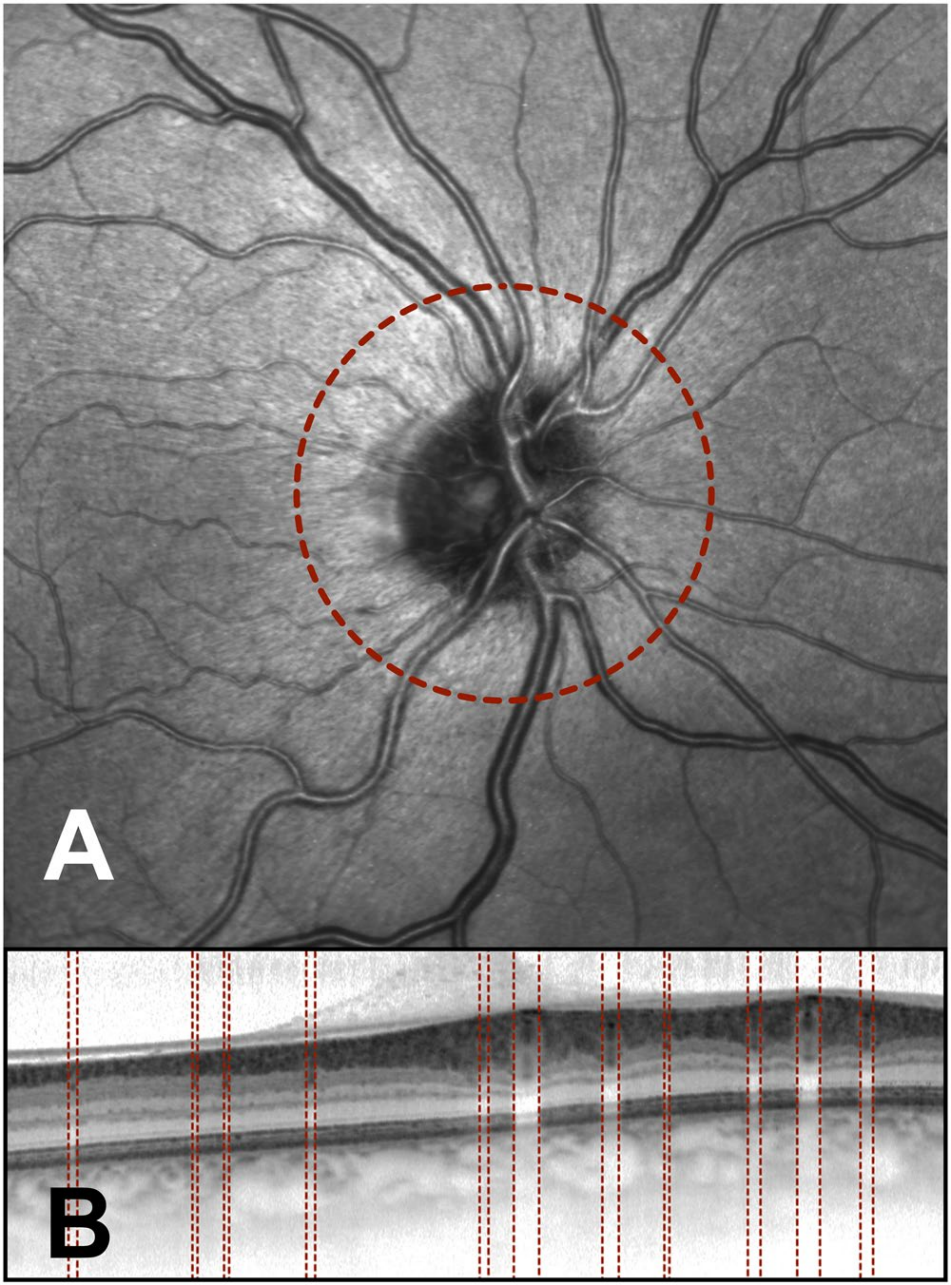
Peripapillary spectral domain optical coherence tomography (SD-OCT) scan (**B**) and the linked confocal scanning laser ophthalmoscope (cSLO) fundus image (**A**) obtained from a subject who had not experienced a myocardial infarction at a young age (<50 years). The peripapillary OCT scanning circle was 3.5 mm in diameter and is shown in the cSLO image (red circle, A). The manually identified vessel borders are shown on the SD-OCT scan (red lines, B).

The SD-OCT system noninvasively obtains high-resolution, microscopic, cross-sectional images of the retina. The device combines conventional OCT technology with cSLO technology. A super-luminescent diode emits a light beam with a wavelength of 870 nm. The SD-OCT has a scan rate of up to 40,000 A-scans per second, a depth resolution of 7 µm, and a transverse resolution of 14 µm. The cSLO utilizes a laser to illuminate the retina and produce real-time, *en face* images. These fundus images are then used by the SD-OCT system as an anatomical reference. Before obtaining SD-OCT scans, the scan circle was centred on the optic disc. The automatic real-time averaging mode, resulted in the achievement of even higher quality. In this study, high-quality SD-OCT scans with at least 50 frame averaging were used to provide images with low noise. Three high-resolution SD-OCT peripapillary scans with 3.5 mm in diameter and linked cSLO images were acquired from each subject. All SD-OCT images were obtained by a single examiner to minimize variability. Additionally, all included scans satisfied the consensus criteria for retinal OCT quality assessments (OSCAR-IB) to improve scan comparability and ensure that only the highest quality OCT images were used^45^. More specifically, any image that did not meet all of the following quality criteria were excluded from analyses: (i) in focus and visible fundus before and during image acquisition, (ii) absence of scan and algorithm failures, and (iii) a continuous scanned layer. A representative peripapillary SD-OCT scan with a linked cSLO image is shown in Fig. 3.

#### Manual classification of retinal vessels in cSLO images

Retinal vessels were manually classified as arterioles or venules because automated classification methods do not yield adequate results, even when colour fundus photographs are used^46^. Determining vessel classification is more complicated on cSLO images than on colour fundus images because cSLO images are monochromatic. Around the optic disc, only vessels that are close to each other can be reliably categorized as arteries or veins because a direct comparison is needed to reliably distinguish between vessel types. The following comparisons were used to categorize vessel: (i) the central light reflex is wider in arteries and smaller in veins^47,48^, (ii) arteries are brighter than veins^48^ and veins are darker and deeper than arteries^47^, and (iii) arteries are 30% thinner than their neighbouring veins^48^.

#### Calculation of the arterial-venous ratio

Vessel diameters were indirectly measured on peripapillary SD-OCT scans using vessel-associated shadowing, as previously described^49^. Vessel diameters were measured indirectly, and their shadow width on the SD-OCT scan was determined, assuming that retinal vessel blood flow attenuates the SD-OCT signal underneath the vessel. The central retinal equivalent was computed from these measurements using formulas established by Knudtson *et al*.^50^, which optimises the popular approach reported by Parr and Hubbard^51–53^. One limitation of the Parr-Hubbard formula is that the number of vessels measured significantly impacts the overall AVR calculation. Knudtson *et al*.^50^ based their formula on the six largest retinal arterioles and venules to overcome this. The following equations were used to calculate CRAE-6 for arterioles and CRVE-6 for venules:

1 $$Arterioles:\hat{W}=0.88\,\ast \,{({w}_{1}^{2}+{w}_{2}^{2})}^{1/2}$$

2 $$Venules:\hat{W}=0.95\,\ast \,{({w}_{1}^{2}+{w}_{2}^{2})}^{1/2}$$

where w_1_, w_2_, and $\hat{W}$ are, respectively, the widths of the narrower branch, the wider branch, and the the estimate of parent trunk arteriole or venule. The AVR is then calculated as the quotient of CRAE-6 and CRVE-6 using the same iterative procedure of combining the largest and smallest vessel in each pairing.

### Statistical analyses

Eyes from each participant were analysed separately (phenotype). Data are presented as mean ± standard deviation. All statistical analyses were performed using a commercially available software package (Prism 7 for Mac OSX; GraphPad Software, Version 7.0a, GraphPad Software, Inc., La Jolla, USA). Two-tailed, paired parametric t-tests were used to test the statistical significance of differences between continuous variables. Correlation analyses were performed using Pearson correlation calculations because data sampled from the populations followed an approximate Gaussian distribution. Statistical significance was defined as p < 0.05.

A two-tailed power analysis was also performed with a significance level (alpha) of 0.05. Because Schuster *et al*.^49^ found an AVR difference of 0.09 between healthy patients and those with hypertensive retinopathy, the power analysis was performed for an AVR difference of 0.09. A post-test power analysis was also performed on the smallest detected difference to verify that observed differences were not insignificant because of relatively small sample size.
